# Predictor of multidrug resistant tuberculosis in southwestern part of Ethiopia: a case control study

**DOI:** 10.1186/s12941-018-0283-8

**Published:** 2018-07-03

**Authors:** Dabesa Gobena, Gemechu Ameya, Kinfe Haile, Getaneh Abreha, Yoseph Worku, Tessema Debela

**Affiliations:** 1 Oromia Regional Health Bureau, Addis Ababa, Ethiopia; 2 Department of Medical Laboratory Science, College of Medicine and Health Sciences, Arba Minch University, P.O. Box: 21, Arba Minch, Ethiopia; 3 Saint Paul Hospital Millennium Medical College, Addis Ababa, Ethiopia; 4 Ethiopia Public Health Institute, Addis Ababa, Ethiopia

**Keywords:** Multi-drug resistance tuberculosis, Predictor, Tuberculosis treatment, Southwest Ethiopia

## Abstract

**Background:**

Curable disease tuberculosis is becoming incurable or difficult to treat due to drug resistance. Multi drug resistance tuberculosis is a major health problem for less developed countries. Development of drug resistance is mainly as result of man related factors and poor lifestyle. Identifying predictors of drug resistance and working on them is the important way of reducing the expansion in high burden countries. Ethiopia is one of TB, TB/HIV, and multi-drug resistant tuberculosis (MDR-TB) high burden country globally. This study was aimed to assess predictor of MDR-TB in southwest part of Ethiopia.

**Methods:**

Unmatched case control study was conducted in case to control ratio of 1:1.2 in southwest part of Ethiopia. The cases were recruited from confirmed MDR-TB patient enrolled on second line treatment in Shenen Gibe Hospital (MDR-TB treatment center of the prefecture) and the controls were recruited from previously TB patients who cured or patient with smear negative at the end of treatment month during the study period in the same area. The data was collected by structured questionnaire by interview and logistic regression analyses were used to identify predictors of MDR-TB. Odds ratios with 95% CI were computed to determine the predictors.

**Result:**

From the total 132 participants about 45% of them were cases. None disclosed tuberculosis infected to relatives [AOR = 3.4, 95% CI (1.2–9.8)], insufficient instruction on how to take anti-TB drug [AOR = 4.7, 95% CI (1.4–14.6)], contact history with MDR-TB [AOR = 8.5, 95% CI (2.9–25.5)], interruption of first-line anti-TB treatment for at list 1 day [AOR = 7.9, 95% CI (2.5–24.9)], and having alcohol drinking habits [AOR = 5.1, 95% CI (1.4–18.7)] were identified predictors for MDR-TB infection in study area.

**Conclusion:**

TB infection disclosure status, insufficient instruction on drug usage, contact history with MDR-TB, interruption of first-line anti-TB drugs, and alcohol drinking habits were identified predictor of MDR-TB case. Therefore, early detection and proper treatment of drug susceptible TB, strengthening directly observed treatment, short-course on daily bases, community involvement, and supporting the patient to intervene identified factors is paramount.

## Background

Tuberculosis resistant to Isoniazid and Rifampicin, with or without resistance to any other drugs are became a major health problem all over the world. Multi-drug resistant tuberculosis is mainly manmade crisis related to multiple factors such as poor drug supply management, inappropriate guideline, none adherence, meager infection control practice, improper drug storage conditions, wrong dose or combination, and lack of information no monitoring of treatment [1, 2]. According to 2016 WHO report, more than half a million people were newly eligible for MDR-TB treatment [3]. The prevalence varies among countries and regions while high prevalence is observed in less developed countries. MDR-TB mostly affected the poor, illiterate, reproductive age group, immune-compromised individuals, and low economic status associated with the social structure of the society [1, 2].

Multi drug resistant tuberculosis cases can be in the acquired or transmitted. Acquired drug resistant TB is the development of drug resistance in patients undergoing treatment with first-line drug while infection with resistant bacteria (transmitted) primarily can be occurred. Resistance of microorganisms is caused by a genetic mutation that makes a drug ineffective. Poor administration or insufficient treatment regimen allows drug resistant mutants to become the dominant strain in infected patient. Studies showed multiple factors that associated with development of the drug resistance in different geographical locations [2, 4–6]. Identifying predominant factors in a given region has substantial role in reduction of mortality and morbidity due to the infection.

Challenges include fear of stigmatization which can discourage patients from presenting for treatment and follow-up care and taking medication in front of others; poor drug adherence and medication-associated side effects were common and contributing to high prevalence of MDR-TB. Any delay in diagnosing MDR-TB also impairs a patient’s prognosis and increase the risks of transmitting the disease within the community. MDR-TB is associated with a two to four fold period of treatment, psychological problems, economic wastage, poor treatment adherence and consequently treatment failure. It is also associated with higher case fatality rates [2].

In the face of the high burden of TB in sub-Saharan Africa, the prevalence of MDR-TB increased due to HIV infection. Tuberculosis is still the major public health problem with high mortality and morbidity in developing counties. According to 2017 WHO report, Ethiopia is among the high burden countries with regards to TB, TB/HIV, and MDR-TB [7]. Currently drug resistance is the major challenge in TB control programs. Whether it is pulmonary of disseminated tuberculosis it has significant health and socioeconomic effect on infected individual as well as for family of the patient. According to the Ministry of Health statistics, tuberculosis is one of the leading causes of morbidity, the fourth cause of hospital admission, and the second cause of hospital death in Ethiopia [8]. However, study related to predictor of multi drug resistance TB not yet conducted. The aim of this study was to assess major predictor of MDR-TB in southwest part of Ethiopia.

## Methods

### Study design and study area

An unmatched case–control study was carried out from August 2016 to April 2017. The study was conducted in Jimma zone, Oromia regional state of Ethiopia at Shenen Gibe hospital. According to the Ethiopian 2007 population and housing survey the projected total population of the zone was 3,214,716 [9]. The zone has 21 rural Districts, two town administrations, one specialized teaching hospital and four primary district hospital, 110 health centers and 503 Kebeles (the smallest administrative structure). According to data from Oromia regional health bureau, Shenen Gibe hospital is found in Jimma town serving as MDR-TB center for southwestern part of the Ethiopia specifically for Jimma zone, Illuababora zone, Gambella region, and Jimma town.

### Study population

The cases were recruited from confirmed MDR-TB patient enrolled on second line treatment in Shenen Gibe Hospital (MDR-TB treatment center) during the study period. The controls were recruited from previously TB patient but declared cure, patients who had completed treatment, and patient with smear negative at the end of treatment month during the study period. MDR-TB patient not on treatment during the study period and seriously ill patients were excluded.

### Sample size and sampling procedures

Epi-Info version 7.2.1 was used to calculate the number of study participant included in the study. Previous study conducted in the Amhara regional state [10] was used to calculate sample size. The estimated sample size was determined based on the following assumptions: confidence interval of 95% at power of 80%, with the ratio of 1:1 (case to control), percent of control exposed 35.3 and percent of cases exposed 61.2 were 122 (61 cases and 61 controls) and 10% non-response rate. Hence, the total sample size was 135.

MDR-TB patients enrolled to second line drug at Shenen Gibe Hospital from 2015 to 2016 were taken as cases and simple random sampling technique was used to include study subjects from the list of MDR-TB patient TB register. While Controls were also selected from randomly selected health facility in the zone (22 health facilities from 110 total health facilities in the zone were selected by lottery method). After selecting health facility, TB register was adjusted for controls meet inclusion criteria and sampling frame was made. Finally controls were randomly selected by simple random sampling technique of lottery method from selected health facility.

### Data collection

A standardized investigators administered questionnaires were prepared in English and converted to local language (Afan Oromo) language by fluent speaker to collect information from the participant and converted back to English to check the reliability of data. Health officers and clinical nurses were recruited for data collection. Secondary data was reviewed from TB and MDR-TB registration books. Patient charts and the data collection format were used to determine and record the patients’ initial TB episode. The main variables included in the study instrument were sex, age, occupation, educational status, marital status, monthly income of the family, patient residence, family size, estimated distance of residence from the health facility, smoking, alcoholism, knowledge regarding MDR-TB, means of transportation to reach the health facility, previous TB treatment, previous known contact with an MDR-TB patient, opportunistic infection, defaulting from treatment, total time of first treatment, adverse effects of anti-TB medication, and prior episodes of anti-TB treatment. Data quality control was checked by reviewing with standard national guidelines of MDR-TB and pre tested in the 5% of similar population who not included in the study.

### Data processing and analysis

Collected data were entered to Epi-Info version 7.2.1 and exported to SPSS version 20.0 for further analysis. Data completeness and consistency was checked by running frequencies of each variable. Analyses of variables were made using descriptive statistics and logistic regression analysis to look into the predictor of MDR-TB. Bivariate followed by multivariable logistic regression analysis was used to identify predictor of MDR-TB infection. Adjusted odds ratios (AOR) and their 95% CI were used to look into the association between the dependent and independent variables.

### Ethical approval and consent to participate

Ethical clearance was obtained from the institutional review board of Saint Paul’s Hospital Mellinium Medical Collage; clearance was also obtained from Oromia regional health bureaus and from each selected health facility. During the study, written informed consents were obtained from all of the participants included in the study. The right of participants to participate voluntary, with draw or jump any questions not wanted to answer were well respected. Confidentiality was maintained at all levels of the study. Oral assent and written informed consent were obtained from each parent of the children less than 16 years old. Patient involvement in the study was voluntary and those who were unwilling and wanted to quit their participation at any stage were informed to do so without any restriction.

### Operational definitions

The following are operational definitions of variables used in the study

- Drinking habit: an individual who has habit drinking alcohol in regular bases.
- Substance intake habits: patterned use of a substance which have stimulant effect.
- Treatment insufficient instruction: anti-TB drug treatment instruction which lacks important information such as how to take the drug, when to the take, and why to take the drug.
- Supervision by health workers: regular check up done by health care works on anti-TB drug treatment.

## Results

### Socio-demographic data characteristics of the participants

A complete response rate of participant was 98% that results in a total of 132 participants included in the study. The case control study was conducted in case to control ratio of 1:1.2 to determine predictor of MDR-TB in southwest Ethiopia. Male participants account 71 (53.8%) out of all study participants with almost equal allocation for case and control. The age range of the participants was from 10 to 70 years while the median age of the respondents was 27.5 years with the mean and standard deviation of 30.2 ± 12.7 with no significant difference between case and control. The majority of participants were in age group from 25 to 34 followed by 15–24 years. About 58% of cases and 40% of control were urban dweller results about half of the overall study participant the study participant. One-third of study participant has no regular income and including this group about 60% of the study participant has less than 500 Ethiopian currencies. Two-fifth of the participants were illiteracy with equal distribution between case and control while the rest had different level formal education as described in the Table 1. More than one-third of study participants were farmers. About a quarter of participants use public transportation while rest traveled on foot as a regular for daily activity (Table 1).

**Table 1:** Socio-demographic data characteristics of the participants

Variables	Cases (n = 59) No. (%)	Controls (n = 73) No. (%)	Total (n = 132) No. (%)
Sex			
Male	36 (61)	35 (48)	71 (53.8)
Female	23 (39)	38 (52)	61 (46.2)
Age			
5–14	5 (8.5)	5 (6.8)	10 (7.6)
15–24	17 (28.8)	20 (27.4)	37 (28)
25–34	20 (33.9)	29 (39.7)	49 (37.1)
35–44	11 (18.7)	10 (13.7)	21 (15.9)
> 45	6 (10)	9 (12.5)	15 (11.4)
Religion			
Muslim	40 (67.8)	53 (72.6)	93 (70.4)
Orthodox	9 (15.2)	18 (24.7)	27 (20.4)
Protestant	7 (11.9)	0 (0)	7 (5.3)
Catholic	3 (5.1)	2 (2.7)	5 (3.8)
Residence			
Rural	25 (42)	44 (60)	69 (52.3)
Urban	34 (58)	29 (40)	63 (47.7)
Marital status			
Married	35 (59)	44 (60.2)	79 (59.8)
Single	19 (32)	17 (23.3)	36 (27.3)
Divorced	4 (6.78)	6 (8.2)	10 (7.6)
Widowed	1 (1.7)	6 (8.2)	7 (5.3)
Monthly income (Eth. Birr)			
< 500	34 (57.6)	45 (61.6)	79 (59.8)
501–1000	13 (32.2)	18 (24.6)	31 (23.5)
1001–1500	6 (10.2)	2 (2.7)	8 (6.1)
> 1500	6 (10.2)	8 (10.9)	14 (10.6)
Educational status			
Tertiary	2 (3.9)	6 (8.2)	8 (6.1)
Secondary	8 (13.5)	8 (10.9)	16 (12.1)
Primary	26 (44)	30 (41)	56 (42.4)
Illiterate	23 (39)	29 (39.7)	52 (39.4)
Means of transportation			
On foot	37 (63)	60 (82)	97 (73.5)
Public transport	22 (37)	13 (18)	35 (26.5)
Occupational status			
Gov’t employee	5 (8.5)	7 (10)	12 (9.1)
Farmers	21 (35.5)	25 (34)	46 (34.8)
House wife	5 (8.5)	14 (19)	19 (14.4)
Merchant	8 (13.5)	6 (8.2)	14 (10.6)
Student	5 (8.5)	9 (12)	14 (10.6)
Daily laborer	9 (15)	8 (11)	17 (12.9)
Others*	6 (10)	4 (5)	10 (7.6)

* Drivers, NGO worker, unemployed

### Medical characteristics and treatment condition of participants

Medical characteristics of the study participants were assessed and presented in Table 2. Out of all the study participants 12% of them were had extra-pulmonary TB while its distribution was about 19% in control group and only about three percent among MDR-TB patients. Nearly 60% of the cases had contact history with MDR-TB cases prior to the onset of their disease. AFB stain detection rate of sputum smear was 42% of the total participants. About 37% of all the MDR-TB cases and 20% of all the control showed cavitations on chest X-ray.

**Table 2:** Medical characteristics of study participants the study participants

Variable	MDR-TB cases (n = 59) Number (%)	Control (n = 73) Number (%)	Total Number (%)
Current TB status			
New	21 (36)	66 (90)	87 (65.9)
Previously treated	38 (64)	7 (10)	45 (34.1)
Site of infection			
Pulmonary	57 (96.6)	59 (80.8)	116 (87.9)
Extra-pulmonary	2 (3.4)	14 (19.2)	16 (12.1)
Contact history with MDR-TB			
Yes	35 (59)	15 (21)	50 (37.9)
No	24 (41)	58 (79)	82 (62.1)
Presence of drug side effect			
Yes	39 (66)	6 (8)	45 (34)
No	20 (34)	67 (92)	87 (65.9)
Hospital admission before on set of disease			
Yes	33 (54)	5 (7)	38 (28.8)
No	26 (46)	68 (93)	94 (71.2)
Previous chronic lung condition			
Yes	24 (41)	2 (3)	26 (19.7)
No	35 (59)	71 (97)	106 (80.3)
HIV status			
Negative	68 (93)	52 (88)	120 (90.9)
Positive	5 (7)	7 (12)	12 (9.1)
Smoking status			
Yes	14 (23.7)	7 (9.6)	21 (15.9)
No	45 (76.3)	66 (90.4)	111 (84.1)
Drinking status			
Yes	19 (32.2)	6 (8.2)	25 (18.9)
No	40 (67.8)	67 (91.8)	107 (81.1)
Diabetic history			
Yes	8 (13.6)	1 (1.7)	9 (6.8)
No	51 (86.4)	72 (98.6)	123 (93.2)

With major proportion of cases, about 34% of all the respondents were previously treated with first-line anti-TB treatment before current treatment. Two-third of the MDR-TB cases were previously treated with anti-TB drug. Of all the MDR-TB cases, 37 and 27% of them had history of treatment failures and relapse respectively. One-third of participant experienced recognizable side effect among all participant and this proportion raised to two-third in MDR-TB cases. According to MDR-TB patient response, regular follow up by health workers was observed in two-third of them and only 57% of the cases were well instructed about anti-TB drugs (Table 2).

Forty-one percent of MDR-TB cases had previous chronic lung condition. Among all study participants, about 9% of them had HIV sero-positive status while 6.8% of them were with diabetic history. Concerning behavioral factors of the respondents, only about 16% of the respondents were smokers while majority of them were among cases. About one-fifth of all the respondents and one-third of the cases had history of drinking alcohol, and nearly half of the respondents had habit of substance intake for recreation (Table 2).

### Predictor of MDR-TB cases among study participants

In bivariate logistic regression analysis, about eight variables that had *p* value less than 0.2 were selected for multivariate analysis. However, among 18 variables that entered into multi variable logistic regression; only five of them particularly no discloser about infection, insufficient instruction on how to take the anti-TB drugs, contact history with MDR-TB, missed anti-TB drug at least for a day, and drinking habit of the participants independently identified as predictor of MDR-TB in study population. On the other hand, smoking habits, gender of the participants, and being pulmonary or extra-pulmonary infection were not significantly associated with development of MDR-TB in multivariate logistic regression.

Those TB patient who do not disclosed their infection status by mycobacterium tuberculosis had about three times more odds of developing MDR-TB as compared to those patient who disclosed their infection to relatives [AOR = 3.4, 95% CI (1.2–9.8)]. Patient who got insufficient instruction about anti-TB drugs had nearly five times more chance of developing MDR-TB than those who got sufficient instruction [AOR = 4.7, 95% CI (1.4–14.6)]. Patient who had contact history with MDR-TB were 8.5 times more likely infected with MDR-TB than their counterparts [AOR = 8.5, 95% CI (2.9–25.5)]. Patients who interrupt previous first-line anti-TB treatment for at list 1 day had eight times more odds of developing MDR-TB [AOR = 7.9, 95% CI (2.5–24.9)]. Having alcohol drinking habits had five times more change of getting MDR-TB in this study [AOR = 5.1, 95% CI (1.4–18.7)] (Table 3).

**Table 3:** Predictor of multi-drug resistant tuberculosis among

Category	Variables	Cases n (%)	Controls n (%)	COR (95% CI)	AOR (95% CI)	P-value
Sex	Male	36 (61)	35 (48)	1.7 (0.8–3.4)	2.4 (0.9–6.7)	0.069
	Female	23 (39)	38 (52)	1	1	
Disclosed about infection	Yes	25 (42.4)	45 (61.6)	1	1	
	No	34 (57.6)	28 (38.4)	2.2 (1.1–4.4)	3.4 (1.2–9.8)	0.021
Sufficient instruction about the drugs	Yes	34 (57.6)	62 (84.9)	1	1	
	Not	25 (42.4)	11 (15.1)	4.1 (1.8–9.4)	4.7 (1.4–14.6)	0.008
Contact history with MDR-TB patient	Yes	35 (59)	15 (21)	5.6 (2.6–12.2)	8.5 (2.9–25.5)	< 0.001
	No	24 (41)	58 (79)	1	1	
Missed anti-TB drug at least for a day	Yes	38 (64)	7 (10)	17.1 (6.6–43.8)	7.9 (2.5–24.9)	< 0.001
	No	21 (36)	66 (90)	1	1	
Drinking habit	Yes	19 (32.2)	6 (8.2)	5.3 (1.9–14.4)	5.1 (1.4–18.7)	0.015
	No	40 (67.8)	67 (91.8)	1	1	
Site of infection	PTB	57 (96.6)	59 (80.8)	6.7 (1.4–31.1)	6.8 (0.9–52.4)	0.066
	EPTB	2 (3.4)	14 (19.2)	1	1	

*PTB* pulmonary tuberculosis; EPTB extra pulmonary tuberculosis

## Discussion

The medical condition and socio-demographic factors were identified during the study and a number of factors were independently identified as predictor of infection or development of multi drug resistant tuberculosis. In our study, about two-third of the MDR-TB cases were previously treated with first-line drug. This indicates that the large proportion of drug resistance may be developed in patient during the first treatment rather than transmitted. In Ethiopia different studies indicated that previous exposure to TB treatment was found to be the most significant risk factors of MDR-TB [11–16]. These may be because of poor adherence to the treatment and lack of supervision by health workers, lack of medication, distance from health facility or because of side effect of medication. Study conducted in urban Lima, Peru showed confirmed acquired resistant was less than transmitted MDR-TB and a large groups were unidentified [17]. This difference between acquired and transmitted drug resistant tuberculosis may be due to different factors such as the awareness and knowledge of proper drug usage, socio-demographic status of patients, behavioral factors, and patient medical conditions.

In our study different factors became associated with MDR-TB infection. Factors such as absence of discloser infection by patients, insufficient instruction anti-TB drug users, contact history with MDR-TB, missed anti-TB drugs at least for a day, and drinking habit were independently identified as predictor of the infection. In current study absence of disclosure of the previous infection by patient had three times more chance of development of MDR-TB than those who disclose their infection status. Similar finding was observed in studies conducted in Addis Ababa, Ethiopia [18], and Nepal [4]. Patients conceal their infection status mostly due to fear of stigma and lack of support from their relatives. This factor may affect the proper use of drug according to prescription.

Proper use of drug according to instruction is crucial to prevent resistance developed from miss use of drug. In our study, patient who got insufficient instruction on how to take anti-TB drugs had about five times more chance of developing MDR-TB than their counterpart. Study conducted in Addis Ababa showed that treatment not directly observed by a health worker was associated with development of multi drug resistant tuberculosis [11]. Insufficient instruction of treatment for patient can lead to adverse effects of anti-TB drugs or drug interactions can make it necessary to modify or discontinue treatment. Adverse reactions to anti-TB drugs can be due to multiple factors and the most important factors are the dose and time of day at which the medication is administered, nutritional status, patient age, preexisting diseases, behavioral factors, and status of major organ function. Therefore, considering these important factors during instruction is important to reduce drug resistance [19].

Having contact history with MDR-TB or other chronic coughers was another predictor of MDR-TB in our study. Similar findings were observed in study conducted in Oromia region of Ethiopia [5], Amhara region of Ethiopia [10], Pakistan [20], Namibia [6], and in Tanzania [21]. These may due to individuals who had close or regular contact had the chance of getting the strain of drug resistance TB since the few load of bacteria can be transmitted aerosol and respiratory droplets as well as other means of contacts.

Drug interruption is important factors for development of drug resistant pathogenic strains. In current study, missed first-line anti-TB drug at list for 1 day was strongly identified predictor of MDR-TB cases. Study conducted in Addis Ababa Ethiopia also showed similar finding that those TB patient interrupt first-line anti-TB drug at list for a day was about thirty more chance of MDR-TB development [11]. A systematic review conducted in Europe also revealed previous treatment was highly associated with development of MDR-TB [22]. According to the Federal Ministry of Health [8], in Ethiopia history of using poor quality TB drugs, treatment in a poorly-performing control program was the major challenge in TB treatment program. Drug interrupting may be in consequence of fear of side effect medication, change in period of treatment, and lack of sufficient informing about the treatment.

So far different study exhibited the role of behavioral factors in development of multidrug resistant tuberculosis. In our study, having history of drinking alcohol was about five times more risk for development of MDR-TB. Study conducted in Nepal [23], and Thailand [24]. Similarly, study conducted in Oromia, and Amhara region of Ethiopia also showed similar findings, in which patients who drink alcohol had more risk of infection or development of MDR-TB [5, 10]. In study conducted in Russia, drinkers had less favorable treatment outcomes. This is associated with problem of adherence to prescribed doses and occurrence of adverse events that requires treatment interruption [25].

## Conclusion

Majority of patients who develop multi drug resistant tuberculosis were on first-line anti-TB treatment. Patients who were not disclosed their infection status, patient who receive insufficient instruction on how take anti-TB drugs, patient who had contact history with MDR-TB, interruption of first-line anti-TB drugs for at list 1 day, and patient who had alcohol drinking habits were independently identified as predictor of MDR-TB cases. Using directly observed treatment, short-course effectively and working on identified factors is mandatory to reduce the development drug resistance.

## Abbreviations

AOR: adjusted odd ratio
CI: confidence interval
HIV: human immunodeficiency virus
MDR-TB: multi-drug resistant tuberculosis
TB: tuberculosis
WHO: World Health Organization
